# IER5 as a promising predictive marker promotes irradiation-induced apoptosis in cervical cancer tissues from patients undergoing chemoradiotherapy

**DOI:** 10.18632/oncotarget.16857

**Published:** 2017-04-05

**Authors:** Yang Liu, Ming Tian, Hui Zhao, Yue He, Fengshuang Li, Xiunan Li, Xinping Yu, Kuke Ding, Pingkun Zhou, Yumei Wu

**Affiliations:** ^1^ Department of Gynecologic Oncology, Beijing Obstetrics and Gynecology Hospital, Capital Medical University, Beijing 100006, P.R.China; ^2^ Department of Radiation Toxicology and Oncology, Institute of Radiation Medicine, Academy of Military Medical Sciences, Beijing 100850, P.R. China; ^3^ National Institute for Radiological Protection, Chinese Center for Disease Control and Prevention, Beijing 100088, P.R. China

**Keywords:** IER5, cervical cancer, radiosensitivity, apoptosis, biomarker

## Abstract

**Purpose:**

To define the role of immediate-early 5 (IER5) gene as a promising biomarker in predicting the radiosensitivity and prognosis of cervical cancer patients receiving cisplatin-based concurrent chemoradiotherapy (DDP-CCRT).

**Results:**

Our investigations found that IER5 level was markedly elevated in cervical cancer patients after being treated with irradiation, which indicated IER5 was closely dose induced. By contrast, the correlation between IER5 and radiosensitivity cannot be confirmed by the present study. The up-regulation of IER5 expression effectively increased cell apoptosis after administration of irradiation (*P* < 0.05). Using an ANOVA model for repeated-measures, we found significant association between the IER5 level and tumor size (*P* < 0.05).

**Materials and Methods:**

Forty-three cervical cancer patients stage II_b_-III_b_ received DDP-CCRT were registered. Biopsy tissues were obtained after administration of irradiation dose of 0 Gy, 2~6 Gy, 10 Gy, 20 Gy, 30 Gy, respectively. The IER5 protein and mRNA levels were measured by immunohistochemistry, western blot and quantitative polymerase chain reaction, respectively; besides, the apoptosis rate was assessed by transferase-mediated dUTP nick end labeling.

**Conclusions:**

Mechanistically, we confirmed that IER5 induced by radiation dose enhanced apoptosis of cervical cancer, was inversely associated with tumor size. In conclusion, our studies indicate target IER5 is improved to be a potential radiosensitizer for developing effective therapeutic strategies against cervical cancer to radiotherapy and a predictive biomarker for radiosensitivity.

## INTRODUCTION

Cervical cancer is the second malignancy leading cause of death for women in the underdeveloped regions [1]. Concurrent chemoradiotherapy is the most commonly preferred treatment modality for patients with advanced cervical cancer [2]. However, the overall prognosis of cervical cancer is still far from satisfactory, with a high incidence of tumor recurrence, the 5-year overall survival rate is only 67% and nearly half (44%) of the patients experience a relapse [3]. As the crowd for radiosensitivity varies widely, so the efficacy and prognosis is different. Therefore, to combat this problem, the molecular profiling of tumor and biomarkers studies need to be pursued to early screen for sensitive groups so as to develop individualized treatments and explore effective radiosensitive targets.

As is shown in the current genomics analyses most cancers derive from mutations in a subset of genes, which highlight the importance of genomic instability on the process of carcinogenesis [4]. The induction of DNA lesions is the predominant cytotoxic mechanism of radiotherapy during cancer treatment. Numerous studies focusing on radiosensitizer were reported, with findings that some gene products improved sensitivity of tumor cells via cell cycle control, apoptosis regulation and DNA damage repair mechanism [5]. Current analyses have revealed a potential role for DNA repair-associated genes/proteins as biomarkers in a variety of cancers, including gynecologic cancer. Some have already been applied to clinical practice, the ribose polymerase (PARP) inhibitor—Olaparib, for instance, demonstrated a remarkable level of success in treatment efficacy (reviewed in [6]).

Notably, IER5 as a member of the slow-kinetics class of immediate-early genes [7] has demonstrated playing an essential role in nuclear response to extracellular signals, particularly with respect to radiation in diverse cancers [8, 9]. Several previous studies have confirmed that IER5 is overexpressed and associated with high apoptosis rate in cervical cancer induced by radiation, alternatively, the high proportion of apoptosis promoted the radiosensitivity conferred by IER5 gene amplification [10]. In view of the relationship between IER5 and clinical pathological characteristics still remains obscure, as well as the debated function of IER5 in cell apoptosis, further information about the dynamics of IER5 response in irradiation presents an opportunity to advance the application of molecular driven therapeutics during radiotherapy in treatment of cervical cancer. Thus, our trial was designed to (i) explore expression of IER5 as a radiosensitive and prognostic marker of patients with cervical cancer undergoing radiotherapy, (ii) evaluate IER5 as a predictor in response to radiotherapy of cervical cancer, and (iii) preliminarily inquiry the role of IER5 in response of apoptotic cell death induced by irradiation in cervical cancer tissue.

## RESULTS

### Up-regulated IER5 expression is significantly correlated with radiation dose

**Figure 1:**
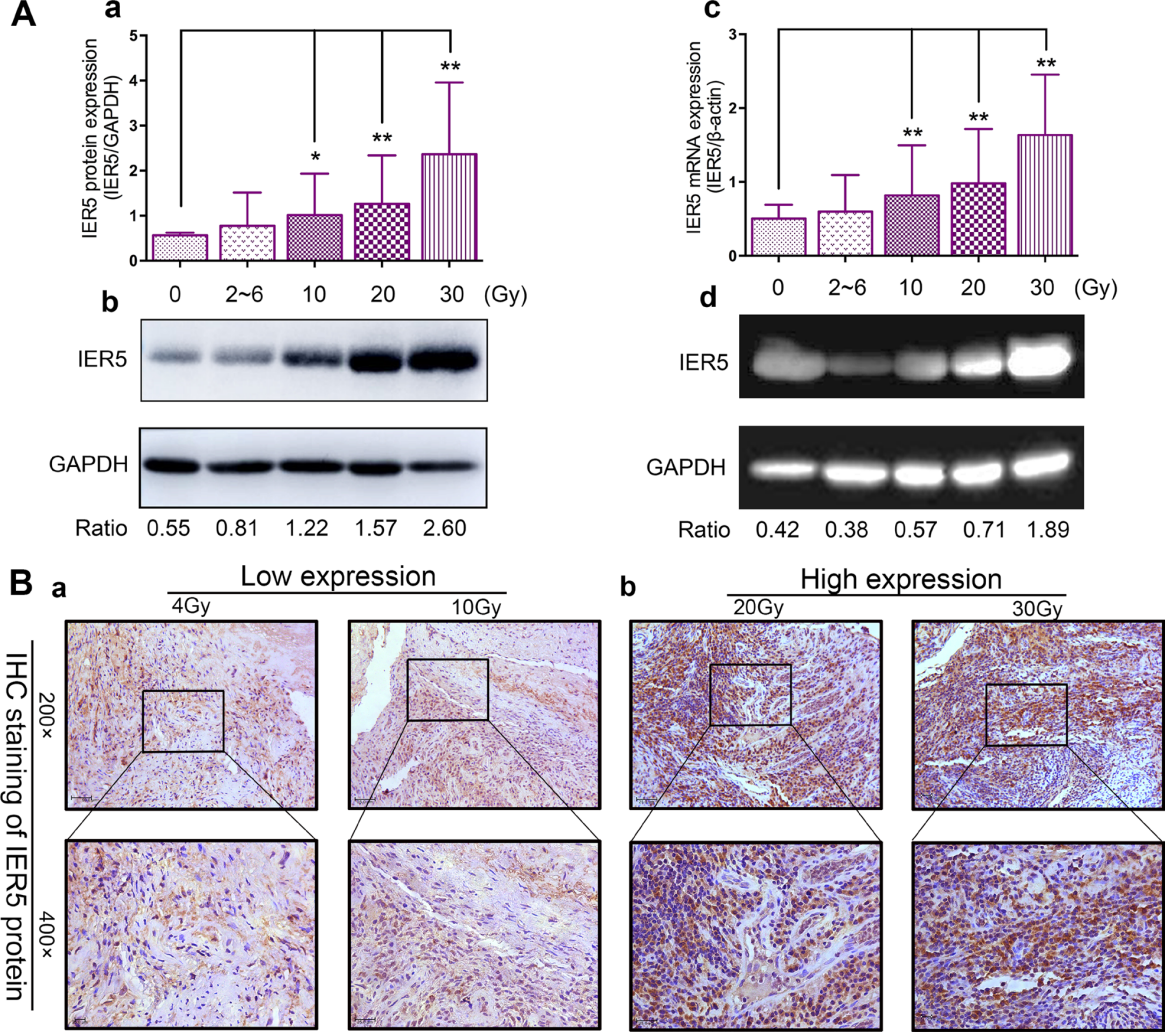
IER5 expression in each group (**A**) IER5 protein expression in human cervical cancer were treated with radiotherapy (radiation dose range from 0 Gy to 30 Gy), were assessed using Western blotting (**Ab**) and RT-PCR (**Ad**). The results are analyzed compare to the untreated control (0 Gy group). (**Ac**) Analyzed by qRT-PCR, IER5 mRNA expression in 10 Gy, 20 Gy and 30 Gy groups were significantly higher than that in 0 Gy group. β-actin was used as the internal loading control. (**Aa**). Western blotting analysis were used, showing the similar results as qRT-PCR analysis. GAPDH protein expression is shown as an internal control. (**B**) Representative images of IER5 protein staining. According to IHC immunostaining determination, patients dichotomized into high IER5 expression group (+2/+3) (**Ba**) and low IER5 expression group (0/+1) (**Bb**). Original magnification: 200 ×, 400 ×. Scale bars: 25.0 μm. Abbreviations: IER5, immediate-early response5; IHC, immunohistochemistry. Each assay was performed at least three times, and the results are expressed as means ± SD. *P < 0.05, **P < 0.01.

Previous quantitative realtime-PCR (qRT-PCR) assay was performed to identify expression of IER5 in cervical cancer biopsies receiving DDP-CCRT. Results showed that IER5 expression was significantly higher after administration of radiation (Figure 1Ac and 1Ad). To confirm the results of qRT-PCR, western blot was performed to determine IER5 expression of the cohort (Table 1),which indicated that the protein expression level of IER5 in ≥ 10 Gy (10 Gy, 20 Gy and 30 Gy) groups was significantly higher than the group prior to therapy, high dose (20 Gy and 30 Gy) groups was significantly higher than low dose (2~6 Gy and 10 Gy) groups and the untreated (0 Gy) group. However, no significant difference was observed between the 0 Gy and the 2~6 Gy group, showing the similar results as qRT-PCR in these matched specimens (Figure 1Aa and 1Ab). Immunohistochemical (IHC) assays of specimen showed IER5 expression was highly located in both nucleus and cytoplasm, and further validated that the expression level of IER5 was significantly higher after administration of radiation (Figure 1Ba and 1Bb). Combined with the above results, conclusion can be drawn that these data proved that up-regulated IER5 expression is significantly correlated with radiation dose.

**Table 1:** The isolation between IER5 and radiation dose (means ± SD)

RT dose (Gy)	IHC (*n* = 33)	qRT-PCR (*n* = 43)	Western Blot (*n* = 43)
0	0.234 ± 0.160	0.503 ± 0.188	0.564 ± 0.057
2~6	0.250 ± 0.669	0.598 ± 0.496	0.772 ± 0.744
10	0.374 ± 0.095^a^	0.818 ± 0.678^ab^	1.015 ± 0.916^a^
20	0.394 ± 0.076^ab^	0.981 ± 0.734^abc^	1.261 ± 1.078^abc^
30	0.576 ± 0.131^abc^	1.637 ± 0.817^abc^	2.364 ± 1.594^abc^
F	42.17	15.117	13.717
*p*-value	< 0.01	< 0.01	< 0.01

Abbreviations: *n*, the number of patients enrolled; IER5, immediate-early response 5; RT, radiotherapy; SD, standard deviation; IHC, immunohistochemistry.

Note: multiple comparison variance analysis test, Compared with the 0 Gy group, ^a^ *p* < 0.05; Compared with the 2~6 Gy group, ^b^ *p* < 0.05;Compared with the 10 Gy group, ^c^ *p* < 0.05; *p* < 0.05 was considered significantly different.

### Patient baseline characteristics and associations of IER5 expression with clinicopathological characteristics

Forty-three cervical cancer patients were enrolled between October 2014 and November 2015. A total of 215 specimens (0 Gy, 2~6 Gy, 10 Gy, 20 Gy and 30 Gy Group: 43 specimens for each) from 43 patients were detected for the expression of IER5 protein. Correlations between IER5 expression and clinicopathological data of cervical cancer patients are shown in Table 2. Overall, all patients completed both external radiotherapy and brachytherapy without interruption as well as received the initially planned dose of concurrent chemotherapy, while no patient discontinued concurrent chemotherapy prematurely due to serious side effects of medical toxicity.

**Table 2:** Correlations between IER5 expression and clinicopathological data of cervical cancer patients in cohort (*n* = 43)

Patients characteristics	Patient number (%)	*p*-value	
		Western blot	qRT-PCR
**Age (years)**		0.109	0.102
<50	18 (42)		
≥50	25 (58)		
**FIGO stage^a^**		0.392	0.295
IIb	31 (72)		
IIIa-IIIb	12 (28)		
**Histological grading**		0.174	0.127
G2	28 (65)		
G3	7 (16)		
Unknown	8 (19)		
**Pathological type**		0.383	0.377
Squamous cell carcinoma	32 (74)		
Adenosquamous & Adenocarcinoma	11 (26)		
**Tumor diameter^a^ (cm)**		0.027	0.017
< 4	8 (19)		
4~5	28 (65)		
< 5	7 (16)		
**Chemotherapy schedule**		0.921	0.881
Cisplatin	15 (35)		
Cisplatin+5-Fu	28 (65)		
**Hb^b^ (g/l)**		0.523	0.579
<110	19 (44)		
≥110	24 (56)		

Abbreviations: IER5, immediate-early response 5; 5-Fu, 5 fluorouracil; Hb, Hemoglobin; G2, Grade2; G3, Grade3.

Note: multiple comparison variance analysis test; ^a^At the start of treatment; ^b^During the intervention period.

**Figure 2:**
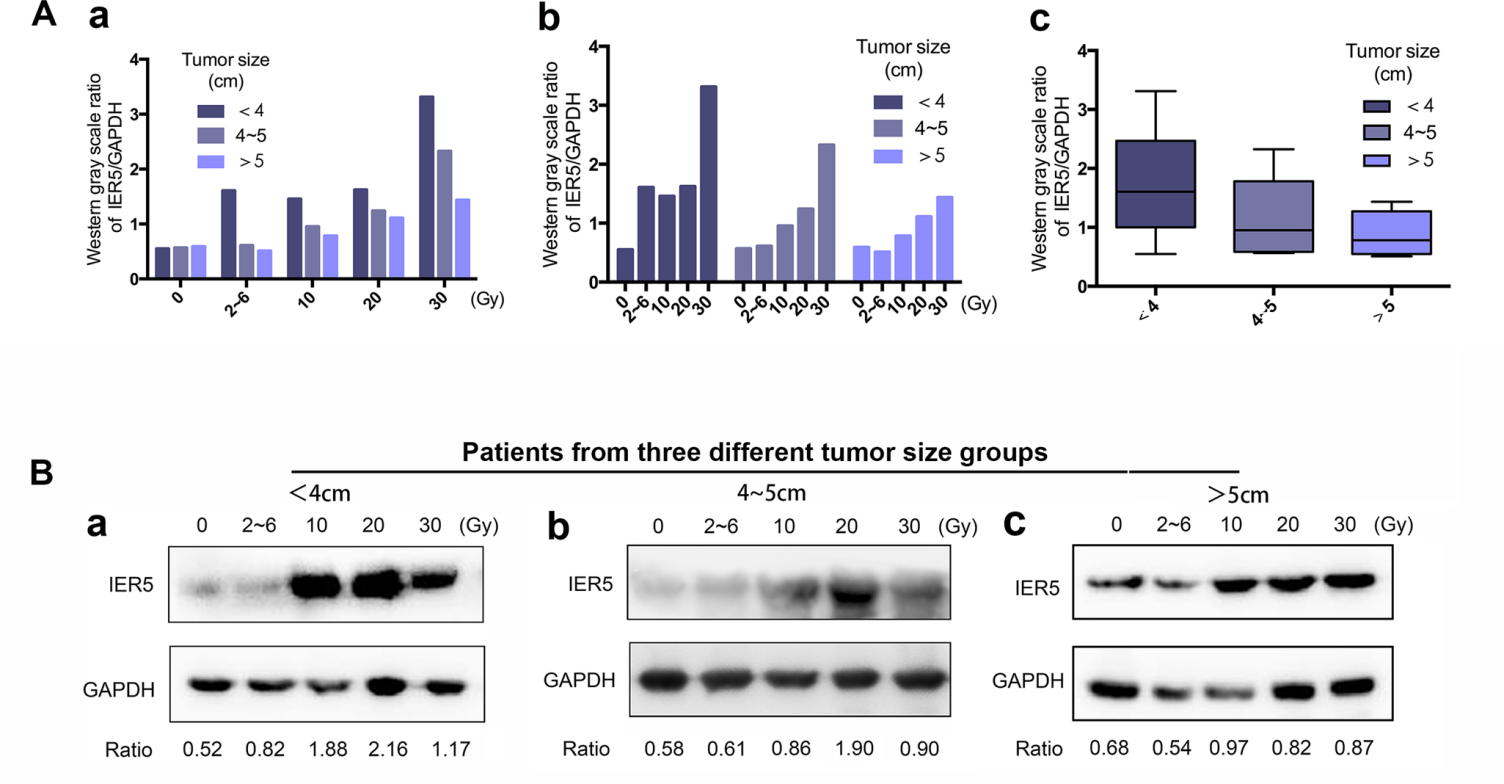
The association between IER5 expression and tumor size (**A**) Analyzed by Western blotting, (**Aa**) IER5 levels of patients in different tumor size groups had no difference. After administration of irradiation dose ranged from 0~30 Gy, (**Ab** and **Ac**) the smaller tumor size group had the higher expression level and the higher variation degree. The IER5 level was counted by Western gray scale ratio of IER5/GAPDH. GAPDH protein expression is shown as an internal control. (**B**) Three typical patients from different tumor size groups (Ba: < 4 cm, Bb: 4~5 cm, Bc: > 5 cm). Abbreviations: IER5, immediate-early response 5.

Next, we analyzed the association of clinicopathological variables with IER5 expression level. Multiple comparison variance analysis test showed. IER5 mRNA protein and mRNA expression (in Western blot and qRT-PCR) was significantly associated with tumor size (*p* = 0.027 and *p* = 0.017). In contrast, IER5 expression was not associated with other malignant clinicopathological variables such as age, FIGO stage, histological grade, pathological type, chemotherapy schedule and anemia index (Hemoglobin) (Table 2). Therefore, we further assessed IER5 expression level among three subgroups of tumor diameter 4 cm, 4–5 cm and > 5 cm group, using the simple effect analysis show that as the tumor diameter of < 4 cm or of 4–5 cm before treatment, the expression of IER5 varied significantly with the radiation dose (*p* < 0.001 and *p* < 0.001), but the tumor diameter of > 5cm was not (Figure 2).

### IER5 activate cell apoptosis, whereas whether radiosensitivity can be promoted by IER5 via facilitating apoptosis route was not proved

Radiation treatment increased the expression of IER5, which promoted cellular apoptotic death in cervical tumors. On the basis of previous research *in vitro*, [11] we hypothesized that IER5 induced by radioactive ray may advance radiosensitivity through activating apoptosis route. Therefore, we assessed whether high expression of IER5 can activate apoptosis route as well as promote radiosensitivity via facilitating apoptosis route.

**Figure 3:**
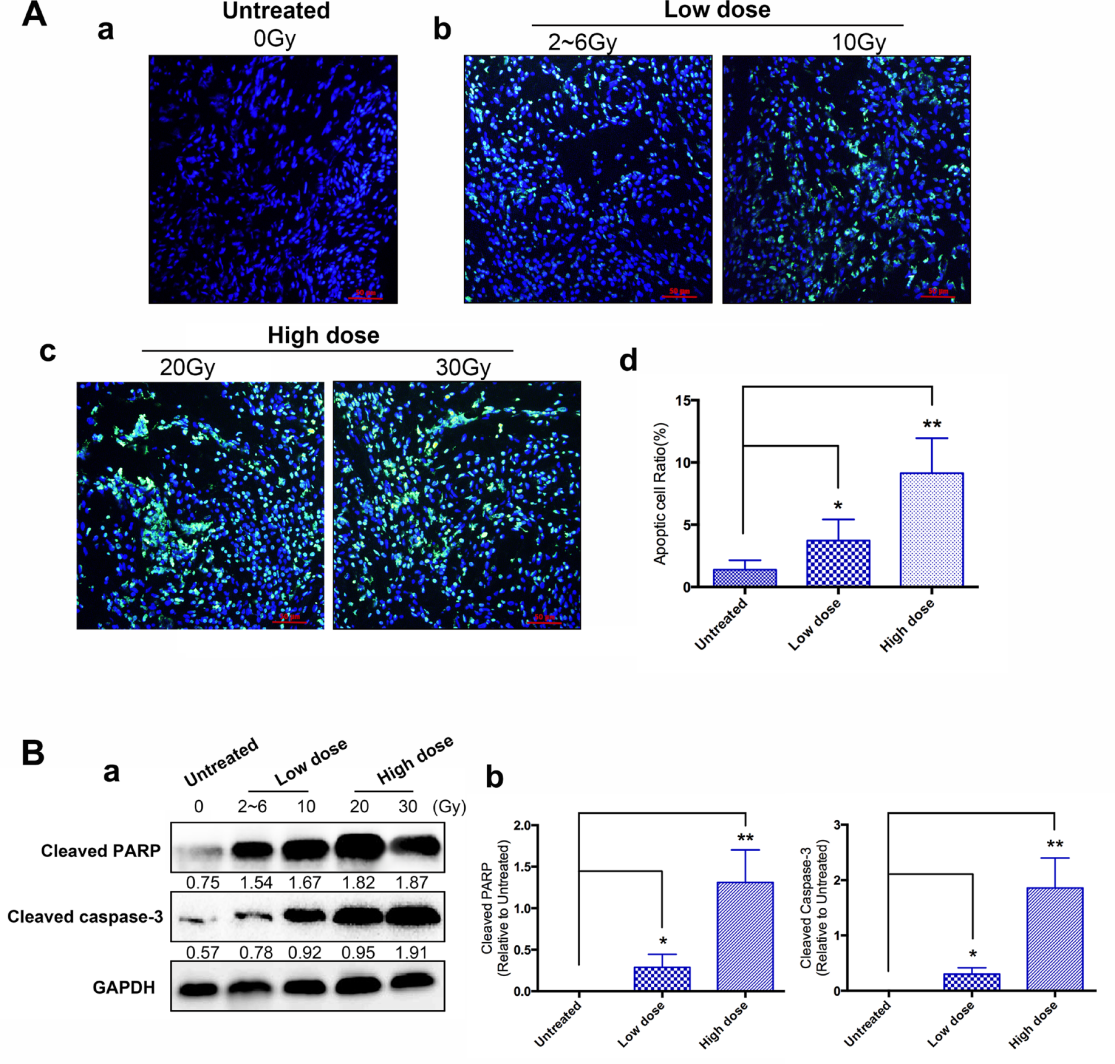
Effects of IER5 upon radiation-induced cell apoptosis in cervical cancer tissues (**A**) To measure the apoptotic cell in radiation-treated cervical cancer tissues, TUNEL assay was performed. Apoptotic cells were visualized into green and nuclei were stained with DAPI (blue). (**Aa**) Untreated group, prior to the radiation treatment. (**Ab**) Low dose group, including the 2~6 Gy and 10 Gy groups. (**Ac**) High dose group, including the 20 Gy and 30 Gy groups. Scale bars are 50 μm. (**B**) (**Ba**) Expression pattern of apoptosis-related proteins (PARP and Cleaved-caspase3) was confirmed by using western blot assay. (**Ad** and **Bb**) Compare with the untreated group. *p < 0.05, **p < 0.01, paired Student's *t-test*.

A total of 60 specimens (0 Gy Group: 20 specimens; Low dose Group: 20 specimens; High dose Group: 20 specimens) from 43 patients were evaluated for apoptosis. Apoptotic cell death was detected by the TUNEL assay and the intensity of image was quantified by ImmunoRatio software. As we see from Figure 3, low dose (Figure 3Ab: 2~6 Gy & 10 Gy Group: 12.80%) and high dose (Figure 3Ac: 20 Gy & 30 Gy Group: 22.15%) of irradiation in cervical cancer tissues showed significantly high apoptosis index compared with the untreated group (Figure 3Aa: 0 Gy Group: 3.11%). In addition, the proportion of apoptosis in high dose groups was significantly higher than the low dose groups. Meanwhile, in order to examine whether IER5 promotes radiation-induced apoptotic changes, the protein levels of cleaved PARP and cleaved caspase-3, which are the active forms of representative apoptotic markers were investigated by using western blot assay (Figure 3Ba). As expected, cleaved PARP and cleaved caspase-3 increased with significant differences between pre-treatment group (0 Gy) and low/high dose of irradiation-treated cervical cancer tissues (Figure 3Ad and Bb). As previous study proved that up-regulated IER5 expression is significantly correlated with radiation dose, high dose led to high IER5 expression (Table 1). TUNEL assay and western blot analysis indicated that IER5 promoted apoptosis, thus suppressing the tumor growth in cervical cancer tissues induced by irradiation.

Forty-three cases of patients with a total of 215 samples were detected by using qRT-PCR assay, as shown in Table 3, which were classified into resistant and sensitive groups according to the RECIST criteria. The IER5 expression in two groups had no significant difference, indicated that there was no significant association between the IER5 level and the radiosensitivity.

**Table 3:** IER5 mRNA expression in sensitive and resistant radiotherapy groups

Group	IER5 mRNA expression					*n*
	0 Gy	2~6 Gy	10 Gy	20 Gy	30 Gy	
sensitive	0.559 ± 0.056	0.835 ± 0.811	1.064 ± 0.013	1.181 ± 1.087	2.446 ± 1.627	33
resistant	0.584 ± 0.061	0.528 ± 0.322	0.825 ± 0.338	1.571 ± 1.051	2.047 ± 1.521	10
*t*	−1.094	1.043	0.653	−0.91	0.626	−
*p*-value	0.281	0.304	0.518	0.369	0.535	−

Abbreviations: *n*, the number of patients enrolled; IER5, immediate-early response 5.

Note: Analyzed by independent sample *t* test.

## DISCUSSION

Cervical carcinoma is a highly heterogeneous disease that radiosensitivity was significantly different between individuals [13]. Collective efforts have been made to identify predictive biomarkers and molecular targeted drugs, while little progress has been made especially in radiotherapy [14]. Therefore, it is necessary to explore the biological mechanism and related interactive factors of the radio-sensitivity [15]. Currently, researchers have observed that IER5, as a promising radiosensitizer for the responses of advanced cervical cancer patients, has been proved plays an important role in the regulation of the cell cycle [16], cell apoptosis [11] and radiosensitivity mechanism [10] in various cells. Although this previously reported IER5 protein was detected in a variety of vitro cell lines, such as HepG2, Hela/Siha and ML-1 etc [11, 17, 18], the previous clinical trials, however, have found limited. Therefore, in this study, taken cervical cancer patients as the research object, the role of IER5 in apoptosis of cervical cancer tissues and its correlation with clinicopathological features were explored.

Ding et al. [9] used qRT-PCR assay to analyze IER5 mRNA expression of human cervical cancer cells (HeLa cell lines) exposed to 60 Co γ-rays, confirmed no remarkable difierence for HeLa response between 2 and 10 Gy.

Moreover, Shi et al. [19] detect IER5 protein in cervical cancer tissue by utilizing immunohistochemistry and Western blot assays, which previously reported its level significantly increased from the ≥ 20 Gy dose groups to < 20 Gy groups. In agreement with these two previous studies, our analysis (immunohistochemistry, western blot and qRT-PCR assays) found that IER5 protein and mRNA expression in < 10 Gy groups (2~6 Gy) increase slightly, contrary to remarkable changes in ≥ 10 Gy groups (10 Gy, 20 Gy and 30 Gy). In this study, we observed that up-regulation of IER5 expression could be promoted by radiation, suggesting a significantly positive association between IER5 and the irradiation dose. IER5 could be a promising predictive biomarker for the responses of cervical cancer patients receiving concurrent chemoradiotherapy.

The relationship between IER5 gene and radiosensitivity had been proved in previous studies, in Ding's research [10], cell growth and survival analyses on IER5-siRNA-Hela cell lines demonstrated that suppression of IER5 potentiated radiation-induced arrest at the G2-M transition, suggesting that suppression of IER5 significantly increased the radio-resistance of HeLa cells. There was no significant difference in the expression of IER5 between the radiation resistant and sensitive groups, which was consistent with the conclusion made by Shi et al. [19] that the expression of IER5 protein was not related to the clinical outcome after receiving radiotherapy. The discrepancy with previous reports could be, we postulate, due to the following circumstances: (i) Owing to the related clinical research samples are relatively small, the conclusion could be greatly influenced by individual differences; (ii) The total follow-up time is relatively short, Overall Survival Rate and Disease Free Survival Rate have not been counted for comprehensive evaluation on the long-term efficacy; (iii) Our further analysis showed no association between IER5 expression and clinicopathological characteristics such as age, FIGO stage, histological grading, pathological type, chemotherapy schedule and hemoglobin. Nevertheless, given the human body is a complex internal environment, uncontrollable factors may exist that influence the effect of radiotherapy and prognosis. Therefore, wider scope of clinical data collection combined with multi-factors comprehensive evaluation would be conducted in our future studies. (iv) A meta analysis of the prognostic value of genes indicates that IER5 significantly connected with poor prognosis of bladder cancer, brain malignant tumor and breast cancer [20]. Ishikawa et al. [21] observed in their experiments that IER5 gene is involved in the regulation of the heat shock cell proliferation. Asano et al. [17] showed that IER5 gene could induce the activation and transcription of HSP gene in HSF1 family. However, in the previous study, HSF1 has been proved to lead to a high degree of malignant tumor and promote tumor growth [22]. Basing on above studies, we speculate that high expression of IER5 is associated with adverse prognosis of cancer patients [23]. Therefore, although the IER5 gene can increase the radiation sensitivity, it can also activate other related oncologic factors leading to the deterioration, finally, results in a variety of clinical outcomes of cervical cancer.

The correlation between tumor size and the prognosis of cervical cancer has been confirmed in previous studies, but the critical value is still controversial in the present literatures [24]. This study showed that there was a strong correlation between the expression of IER5 and the tumor size induced by irradiation, and the smaller tumor size of cervical cancer tissues, the greater variation of the IER5 expression. These data were consistent with previous reports that IER5 was implicated in cancer progression, especially the mechanism of tumor cell growth. Kudaka et al. [25] demonstrated that tumor size can predict prognosis of cervical cancer patients receiving radiochemotherapy to some extent, suggesting an internal relationship between tumor size and cell hypoxia, which hinted that IER5 may be associated with hypoxia related factors and prognosis of cervical cancer.

In the present study, TUNEL technique was performed to assess the apoptosis of cervical cancer tissues from patients undergoing chemoradiotherapy. In the observation, we identified the proportion of apoptosis ratio was lower in the untreated group (0 Gy), followed by significant increases in cervical cancer tissues of the low dose (2~6 Gy and 10 Gy) and high dose (20 Gy and 30 Gy) groups after irradiation. The high proportion of apoptosis in cervical cancer tissues after high dose of radiation could be explained by the fact that IER5 gene may have interactions with other factors through the activation of complicated molecular pathways that could lead to cell apoptosis, thereby affecting the effect of radiotherapy and prognosis of the disease. Yang CJ et al. [11] have also reported that the overexpression of IER5 could inhibit the survival of tumor cells by enhancing the irradiation-induced apoptosis induced by exposure to radiation or cisplatin in human hepatocellular carcinoma cells. Nevertheless, owing to the deficiency of follow-up time, the existing data showed IER5 expression in radio- sensitive and resistance group had no significant difference, which could be a limitation of our study, and long-time follow-up is desirable.

Above all, future work is still urgently required to delineate the secretory mechanisms of IER5 and how it exerts functions in the cell apoptosis. IER5 may be a potential predictive biomarker for the patients with cervical cancer receiving radiotherapy, supporting the pursuit of clinical significance of IER5.

## MATERIALS AND METHODS

### Patient material

From October 2014 to November 2015, a total of 43 confirmed cervical cancer patients (FIGO stage II_**b**_-III_b_) aged between 27 and 72 years old (median, 51)who haven been treated in the Department of Gynecological Oncology, Beijing Obstetrics and Gynecology Hospital, Capital Medical University, China were enrolled. Patients were staged according to FIGO staging 2009 Edition. Only the patients who conformed to the eligibility criteria as follows were selected: i) patients were diagnosed with cervical carcinoma by biopsy without history of other malignancy or cancer therapy; ii) patients had Karnofsky Performance Status (KPS) ≥70; iii) patients had no other serious complications; iv) patients successfully finished the whole course of radiotherapy, with no serious adverse reactions happened during the period. Exclude patients who had serious complications due to other critical diseases, in addition, patients whose clinical information is adequate or unable to be followed up should be removed from the cohort.

### Study design and treatments

External-beam radiation therapy was administered using Cobalt-60 teletherapy machine. Minimum margins were the upper margin of L4-5 (superiorly), the lower margin was the lower edge of obturator (inferiorly), and 1.5–2.0 cm beyond lateral margins of true bony pelvis. All patients underwent external-beam RT with a daily fraction of 180–200 centigrays (cGy), 5 fractions per week. When the biologically effective dose cumulated to 20–30 grays (Gy), a four-field box technique (anteroposterior, posteroanterior and two lateral fields) was delivered, giving a dose of 180–200 cGy, 4 fractions per week. Meanwhile patients were subjected to Iridium-192 based intracavitary brachytherapy, 600 cGy weekly. The whole pelvic irradiation dose was 40–50 Gy in 20–25 fractions and the cumulative biologically effective dose at point A was 36–42 Gy in 6–7 fractions. All patients received radiotherapy concomitantly administered with platinum-based chemotherapy, i.e., cisplatin regimen (cisplatin 40mg/m2 weekly for a total of 3–6 times) or cisplatin united 5-fluorourac regimen (cisplatin 20mg/d+ 5-fluorourac 1g/d, for 5 days every 3 weeks for a total of 2 times). The entire treatment protocol was devised according to the NCCN guidelines.

Fresh cancer tissues were collected and immediately transferred to liquid nitrogen (−196°C) after resection, then laterly long-term maintained at −80°C as a backup for test. The 4–5 mm3 cervical cancer biopsies were examined by two chief physicians from Department of Pathology. Serial biopsy samples before and in the middle of radiotherapy (receiving a radiation dose exceeding < 10 Gy, 10 Gy, 20 Gy and 30 Gy, respectively) were obtained from the patients, which were analyzed for IER5 by performing immunohistochemistry, western blot and qRT-PCR assay. Clinical samples used in this study were approved by Beijing Obstetrics and Gynecology Hospital affiliated Capital Medical University Ethics Committee and written informed consents were obtained from all participants. The detailed patient clinicopathologic characteristics are summarized in Table 2.

### Follow-up and outcome measures

After completion of treatment, telephone and examinations follow-up were conducted every 3 months for the first year, and at 6-month intervals thereafter. Patients were followed up regularly with gynecological examination, blood and urine routine, liver and kidney function, tumor marker, chest X-ray, pelvic and upper abdominal ultrasound or contrast enhanced CT, if the patient was suspected of distant metastasis, a PET/CT was necessarily delivered. All these patients were followed until the deadline of July 31, 2016 or death. Overall survival was calculated in months from the diagnosis until the date of death, last known to be alive, or the study closing date. The median follow-up time in this study was 13 months (range: 8–21 months) measured from the onset of radiotherapy.

According to Response Evaluation Criteria in Solid Tumors (RECIST v1.1.) [12], antitumor activity was determined 6 to 8 weeks after RT completion based on computed tomography (CT) and magnetic resonance imaging (MRI). Tumor response was defined as: Complete Response (CR) when there was disappearance of all lesions; Partial Response (PR) if there was ≥ 30% reduction in the sum of lesion size; Progressive Disease (PD) if there was at least a 20% in the sum of lesion size or appearance of one or more new lesions; Stable Disease (SD) when neither sufficient shrinkage to qualify for PR nor sufficient increase to qualify for PD. Up to the date of follow-up, patients with CR and PR were grouped in the class of radio-sensitive whereas patients with SD and PD were considered radio-resistant.

### Immunohistochemical staining

The streptavidin-peroxidase (S-P) immunohistochemical method (IHC) was performed using a UltraSensitive S-P kit (Boster Bioscience Co., Hubei Province, China) to detect the expression of IER5. Specimens were fixed in 10% formalin solution, and than paraffin-embedded. The sliced 4 μm paraffin embedded sections were deparaffinized with xylene and dehydrated by graded ethanol. The antigen retrieval for IER5 staining was performed by treatment with sodium citrate buffer (10 mM, pH 6.0), followed by samples staining with a microwave oven at 600 W in citrate buffer pH 6.0 for 15 minutes. IHC staining were performed, strictly in accordance with the kit instructions, by using rabbit polyclonal antibodies to IER5 (1:100; NBP1–85935, Novus Bioscience Co., CO, US) as the primary antibodies, correspondingly, using Goat anti-Rabbit IgG (H+L), HRP conjugate (1:250; 31460, Thermo Fisher Scientific Co., WI, US) as the secondary antibody. Images were acquired on a Leica DM 4000B microscope coupled to a Leica DC500 camera (Leica, Wetzlar, Germany) using the Leica Quantimet Q550 Imaging Systems.

### The positive criteria and IHC evaluation

The positive criteria: cells with yellow-brown granules in the cellular nucleus and cytoplasm deeper staining than the background were judged as positive cells (Figure 1B). Five randomly selected discontinuous fields were evaluated under high-power magnification (× 400) in a blinded fashion.

### Qualitative analysis

Judging according to the positive criteria, the percentage of positive cells was count. Determination (judging according to the percentage of positive cells, specimens were classified into four categories): (−), specimens with a percentage of positive cells ranging from 0% to 9%; (1+), specimens with a percentage of positive cells ranging from 10% to 49%; (2+), specimens with a percentage of positive cells ranging from 50% to 79%; (3+), specimens with a percentage of positive cells greater than or equal to 80%. Score 2+/3+ was defined as high expression, while score 0/1+ as low expression.

### Quantitative analysis

The integrated optical density (OD) value of positive tissue staining was measured and analyzed using a computer- assisted genuine color image analysis system (ImagePro-Plus v6.0). The ratio of positive area to total area presented the relative amount of the target substance expression.

### Total RNA extraction and quantitative real-time PCR

Total RNA was isolated from fresh tissues using the TRIzol^®^ (Sigma-Aldrich, Darmstadt, Germany) isolation protocol following manufacturer's instructions. All the experimental vessels were treated with 0.1% diethy pyrocarbonate (DEPC). The RNA concentration and purity were determined by NanoDrop 2000c (Thermo Fisher Scientific Co., WI, US) at 260/280 nm. According to the ReverTra Ace kit (TOYOBO Co. Ltd., Osaka, Japan) specification for the synthesis of cDNA template. Reverse transcription was done using the CFX96 Touch Real-Time PCR System (Bio-Rad Laboratories, Hercules, CA) with SYBR Green^®^ PCR RealMasterMix kit (TIANGEN Bioscience Technology Co. Ltd., Peking, China) in accordance to manufacturer's instructions. Sequences of primers were available on request to evaluate the relative gene expression levels as shown in Table 4. As a housekeeping gene, β-actin was used to normalize for RNA loading. The reactions were carried out in 20 μl of final volume, with 1 μg DNA-free RNA and 50 ng primers. Quantitative PCR was performed under standard cycling conditions: denaturation at 95°C for 1 min, then 40 consecutive cycles at 95°C for 30 sec (denaturation), 60°C for 30 sec (annealing) and 72°C for 45 sec (extension). Results were collected and analyzed with MJ Opticon Monitor software Version 3.1 (Bio-Rad Laboratories, Hercules, CA). The data were analyzed using the comparative Ct method (2−ΔΔCT) according to the Perkin Elmer Instruction Manual.

**Table 4:** Sequences of primers

Gene name	Forward primer (5′→ 3′)	Reverse primer (5′→3′)
IER5	GGACGACACCGACGAGGAG	GCTTTTCCGTAGGAGTCCCG
β-actin	GCGCGGCTACAGCTTCA	CTTAATGTCACGCACGATTTCC

Abbreviations: IER5, immediate-early response 5.

Note: β-actin, used as an internal standard for quantitative mRNA measurement.

### Western blot assay

Frozen tissue samples were grinded into powder form with a mortar and pestle under liquid nitrogen. After RIPA lysis buffer (Thermo Fisher Scientific Co., WI, US) including protease inhibitor mixture (Roche, Mannheim, Germany) was added, tissue cells were lysed on ice for 15 min. The lysates were cleared by centrifugation (14,000 rpm) at 4°C for 5 minutes, and supernatants were collected as protein samples. After total protein extraction, the concentration and purity of lysates were determined by using a NanoDrop 2000c (Thermo Fisher Scientific Co., WI, US) ultra micro spectrophotometer. The supernatants with 6× loading buffer (5:1; volume/ volume) were boiled in water bath for 5min,before subjected to 10% sodium dodecyl sulfate (SDS) polyacrylamide gels and transferred onto nitrocellulose membranes (Bio-Rad Laboratories, Hercules, CA). Nonspecific binding sites were blocked (1 h, room temperature) with 5% non-fat milk made in TBS-T (TBS buffer solution containing 0.05% Tween-20). Blots were then incubated at 4°C overnight with goat polyclonal anti-IER5 (1:500; ab59133, Abcam). The relative protein levels were calculated based on GAPDH as the lane-loading control. The membranes were then incubated with the respective secondary antibody (1:1000; Santa Cruz) for 60 minutes at room temperature after three 10-minute washes with TBS-T. For visualization, an enhanced chemiluminescence kit (ECL, Thermo Fisher Scientific Co., WI, US) plus a high-performance chemiluminescent image analyzer (ImageQuant LAS 500, GE Healthcare Ltd., Buckinghamshire, UK) was applied to the membrane. The intensities of bands were quantified by using Image analysis software (Quantity-One v4.3.0, Bio-Rad Laboratories, Hercules, CA). The relative protein content was represented through the gray value ratio of IER5 protein bands/GAPDH protein bands.

### TUNEL assay

The apoptosis of the cervical cancer tissues were analyzed by terminal deoxynuclotidyl transferase-mediated deoxyuridine triphosphate nick-end-labeling (TUNEL) assay by using TUNEL BrightGreen Apoptosis Detection Kit (Vazyme, Ltd., Nanjing, China), according to the manufacturer's instruction. In brief, after deparaffinization, rehydration, and treatment of a series of graded alcohols (100%, 90%, 80%, 70%), the slides were permeabilized with 20 μg/ml proteinase K for 10 min at room temperature, followed by washing with PBS-T. The tissue sections were then incubated with TUNEL reaction buffer in a 37°C humidified chamber for 1 h, and then incubated with DAPI at room temperature. Stained apoptotic cells were examined under a fluorescence microscope. The intensity of image was quantified with ImagePro-Plus 6.0 software.

### Statistical analysis

Data were analyzed using the statistical software SPSS Version 20.0 (IBM Corp., Armonk, NY, USA). Repeated measures ANOVA was used to examine the effects of age, FIGO stage, histological grading, pathological type, tumor size, chemotherapy schedule and their interactions on IER5 level. Comparisons between radiation sensitive and resistant group for statistical significance were performed with an independent-sample *t* test. Bivariate correlations between study variables were calculated by Pearson's correlation coefficients. Differences between variables of apoptosis rate were assessed by the Chi-square test. All results took α = 0.05 (bilateral) as test standard.
